# Clinical and ocular abnormalities in DEGCAGS syndrome—Developmental delay with gastrointestinal, cardiovascular, genitourinary, and skeletal abnormalities

**DOI:** 10.1002/mgg3.2329

**Published:** 2023-11-28

**Authors:** Syed M. Ali, Dua A. AlMasri, Carlos E. Prada, Doris Lin, Thomas M. Bosley, Igor Kozak

**Affiliations:** ^1^ Moorfields Eye Hospital Abu‐Dhabi Abu Dhabi UAE; ^2^ Mohammed Bin Rashed University Dubai UAE; ^3^ Danat Al Emarat Hospital Abu Dhabi UAE; ^4^ Division of Genetics, Birth Defects & Metabolism Ann & Robert H. Lurie Children's Hospital of Chicago Chicago Illinois USA; ^5^ Department of Pediatrics Feinberg School of Medicine of Northwestern University Chicago Illinois USA; ^6^ Department of Radiology and Radiological Science Johns Hopkins University School of Medicine Baltimore Maryland USA; ^7^ The Neuro‐Ophthalmology Division, The Wilmer Eye Institute Johns Hopkins University Baltimore Maryland USA

**Keywords:** developmental delay with gastrointestinal, cardiovascular, genitourinary, and skeletal abnormalities (DEGCAGS syndrome), eye, retina, ZNF699 gene

## Abstract

**Purpose:**

To describe clinical and ocular abnormalities in a case of Developmental Delay with Gastrointestinal, Cardiovascular, Genitourinary, and Skeletal Abnormalities (DEGCAGS syndrome).

**Methods:**

A clinical report.

**Case description:**

An infant born to a consanguineous Middle Eastern family who was delivered by cesarean section because of in utero growth restriction, premature labor, and breech presentation. Post‐partum medical problems included hypotension, generalized hypotonia, bradycardia, apnea requiring resuscitation and positive pressure ventilation, facial dysmorphia, skeletal malformations, and disorders of the gastrointestinal, immune, urinary, respiratory, cardiac, and visual systems. The family reported that a previous child had severe hypotonia at birth and was given the diagnosis of hypoxic ischemic encephalopathy; that child remains on a ventilator in a chronic care facility. Our patient was found to be homozygous for a novel pathogenic missense variant in theZNF699 zinc finger gene on chromosome 19p13 causing a syndrome known as Developmental Delay with Gastrointestinal, Cardiovascular, Genitourinary, and Skeletal Abnormalities (DEGCAGS syndrome). We review this variable syndrome, including abnormalities of the visual system not described previously.

**Conclusions:**

We describe the 15th child to be presumably identified with the DEGCAGS syndrome and the first individual with homozygous missense variants in the ZNF699 gene who had complete clinical examination and detailed retinal imaging.

## Graphical abstract

Our patient was found to be homozygous for a novel pathogenic missense variant in the ZNF699 zinc‐finger gene on chromosome 19p13 causing a syndrome known as developmental delay with gastrointestinal, cardiovascular, genitourinary, and skeletal abnormalities syndrome. We review this variable syndrome, including abnormalities of the visual system not described previously.

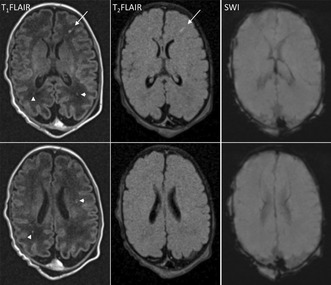

## 1 INTRODUCTION

In 2005, Scholz et al. (2005) first described a novel gene they named *hangover* that was essential in the development of alcohol tolerance in Drosophila melanogaster. The human ortholog of this gene is now known as ZNF699 on chromosome 19p13, which encodes a large zinc‐finger protein, suggesting a molecular role in nucleic acid binding (Fagerberg et al., 2014) and in the development of alcohol tolerance in humans. (Ali et al., 2015; Riley et al., 2006) The gene was not known to be associated with any human genetic disease until 2021, when Bertoli‐Avella et al. (2021) used whole genome/exome sequencing in an effort to identify possible genetic causes of neurodevelopmental delay and/or intellectual disability (ID) of unknown etiology in a group of 33,000 patients. They recognized six possible novel gene‐disease associations linking five different homozygous frameshift variants due to insertions or deletions of ZNF699 to a syndrome they termed developmental delay with gastrointestinal, cardiovascular, genitourinary, and skeletal abnormalities (DEGCAGS syndrome; OMIM 619488).

Bertoli‐Avella et al. (2021) identified 13 children from 12 consanguineous families who had DEGCAGS syndrome that proved to be variable in presentation and sufficiently severe that one evaluated child died prior to their report and eight siblings of the identified children died in infancy (possibly because of the same syndrome). All of the children from their study had a variable phenotype including neurodevelopmental delay, hypotonia, and developmental abnormalities of the heart, gastrointestinal system (intestinal atresia, pyloric stenosis, gastroesophageal reflux, and hepatosplenomegaly), genitourinary system (renal hypoplasia, cryptorchidism, chordee, hypospadias, and ambiguous genitalia), and skeletal system (preaxial polydactyly, absent thumbs, and syndactyly).

In 2022, Biela et al. (2022) were the first to clinically consider the diagnosis of DEGCAGS syndrome in an infant prior to confirming the diagnosis with genetic testing. Their patient had unrelated parents and an older sibling who died in utero at 40 weeks gestation for unclear reasons. Pregnancy and delivery of the child were complicated by intrauterine growth retardation, polyhydramnios, a single umbilical artery, and fetal ileus, and shortly after birth the child was noted to have facial dysmorphism, micrognathia, and duplicated right thumb. The child was transferred to the newborn intensive care unit and on Day 5 underwent an extensive short bowel resection due to multilevel bowel obstruction caused by bowel atresia. She had global hypotonia (with normal deep tendon reflexes) and motor delay, and her early life was marked by several severe infections (SARS‐CoV‐2 and three episodes of bacterial sepsis) precipitating respiratory failure that required a tracheostomy. Whole‐exome sequencing documented compound heterozygous nonsense mutations of the ZNF699 gene (both causing premature termination) with one mutation confirmed in each parent.

The patient reported here is the second child to have the clinical diagnosis of DEGCAGS syndrome considered prior to genetic testing. She may also be the first child with this multisystem, variable syndrome to have a detailed ophthalmologic evaluation with retinal angiography published.

## 2 CLINICAL DESCRIPTION

The child reported here was born to consanguineous parents of Middle Eastern origin who had no major medical problems. The parents have a previous child who at birth was thought to have hypoxic encephalopathy. That child remains ventilator dependent in a long‐term care facility and has not been carefully evaluated either clinically or with whole‐genome sequencing due to parent's refusal. Serologic screening of the mother was unremarkable.

The proband is the second child of this family. The child was conceived naturally, and serial antenatal ultrasound studies showed a single viable fetus with signs of growth restriction and placental insufficiency. The child was delivered by emergency cesarean section at 37 weeks gestation due to breech presentation, intrauterine growth restriction, and preterm labor. At birth, the baby was flaccid, apneic, and bradycardic (60 bpm) with a dusky color. Somatic stimulation, suctioning, and positioning of the airway did not improve respiration, so intermittent positive pressure ventilation was initiated with gradual improvement in heart rate, color, and effort of breathing. Apgar scores were initially low (4, 7, and 8 at 1, 5, and 10 min); birth weight was 1.81 kg with head circumference of 31 cm and a body length of 43 cm. A fracture of the right femur was present at birth and was treated conservatively by splinting.

He developed neonatal jaundice that was treated with phototherapy, and anemia was treated with packed red blood cell transfusions. He had multiple episodes of aspiration and failed to wean off ventilatory support; therefore, at the age of 75 days, he received a tracheostomy and gastrostomy tube with fundoplication. The child was hemodynamically stable, even though a patent ductus arteriosus was noted on screening echocardiogram. He had good urine output, but testes were undescended bilaterally. He was evaluated several times for possible sepsis and treated with antibiotics despite negative cultures.

**FIGURE 1:**
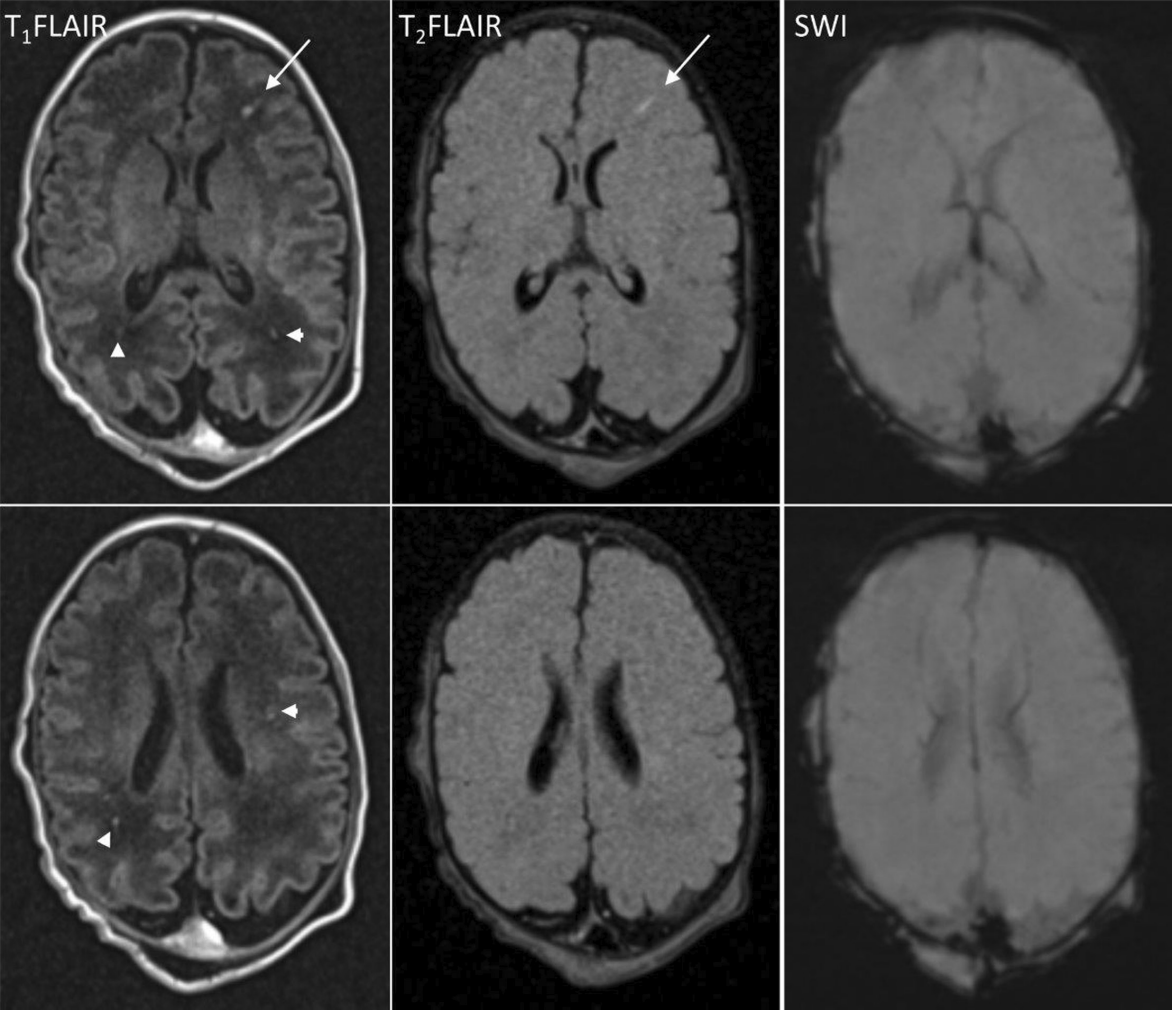
Brain MRI at 8 days of life. Axial T1FLAIR (fluid‐attenuated inversion recovery), T2FLAIR, and SWI (susceptibility weighted imaging) from two different slices at the level of the basal ganglia (top panel) and body of the lateral ventricles (bottom panel) show multiple scattered punctate/small signal abnormalities in the deep white matter bilaterally, without associated susceptibility artifact, suggestive of non‐hemorrhagic white matter injury. Note that a linear focus is well depicted on T2FLAIR (arrow) while many other small lesions are only visualized on T1FLAIR (arrowheads), possibly reflecting different ages of injury. None of these were demonstrated on diffusion‐weighted sequence (not shown), and also not depicted on SWI.

**FIGURE 2:**
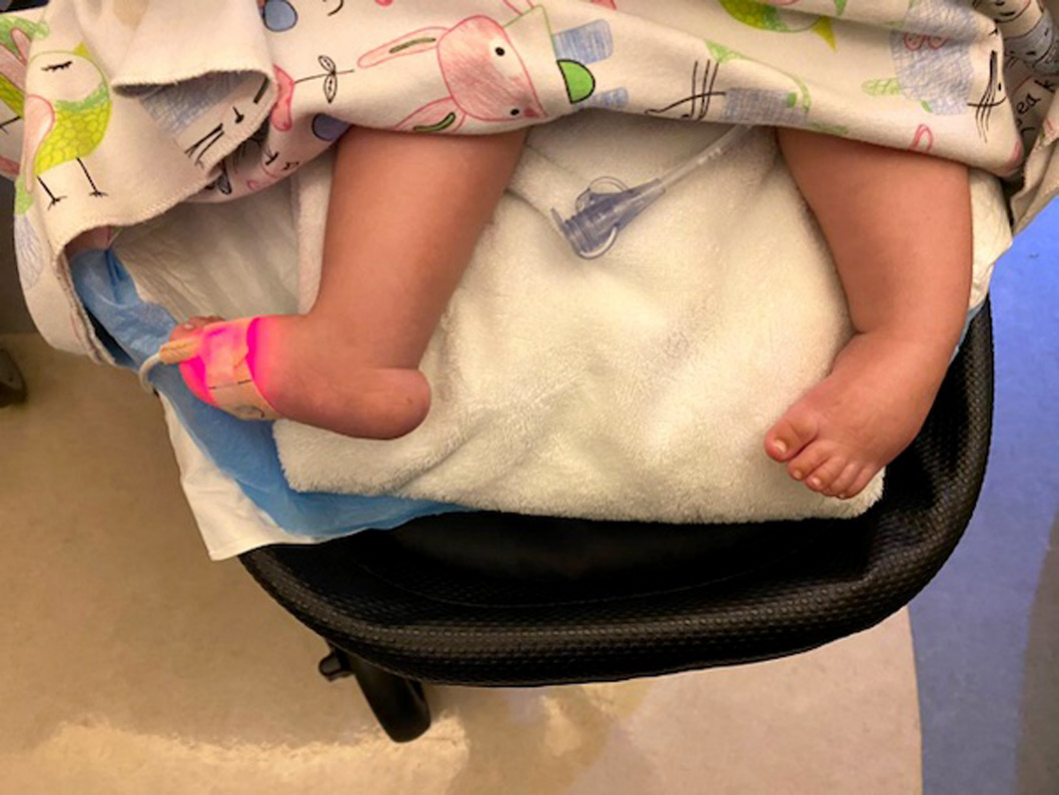
External photograph of the feet demonstrates bilateral talipes equinovarus, congenital vertical talus bilaterally, and rocker bottom feet.

The child had striking diffuse hypotonia at birth with very little movement of any part of his body except his eyes and with no evidence of spasticity or hyperreflexia. MRI scan showed non‐hemorrhagic punctate white matter densities without anatomic brain abnormalities striking enough to explain his lack of mobility (Figure 1). Dysmorphology examination revealed down‐slanting eyes bilaterally, posteriorly rotated ears bilaterally, pectus excavatum, and bilateral single palmar creases. He had bilateral talipes equinovarus, congenital vertical talus bilaterally, rocker bottom feet (Figure 2), long fingers, contractures of both elbows and both knees, and syndactyly of the third and fourth toes bilaterally.

**FIGURE 3:**
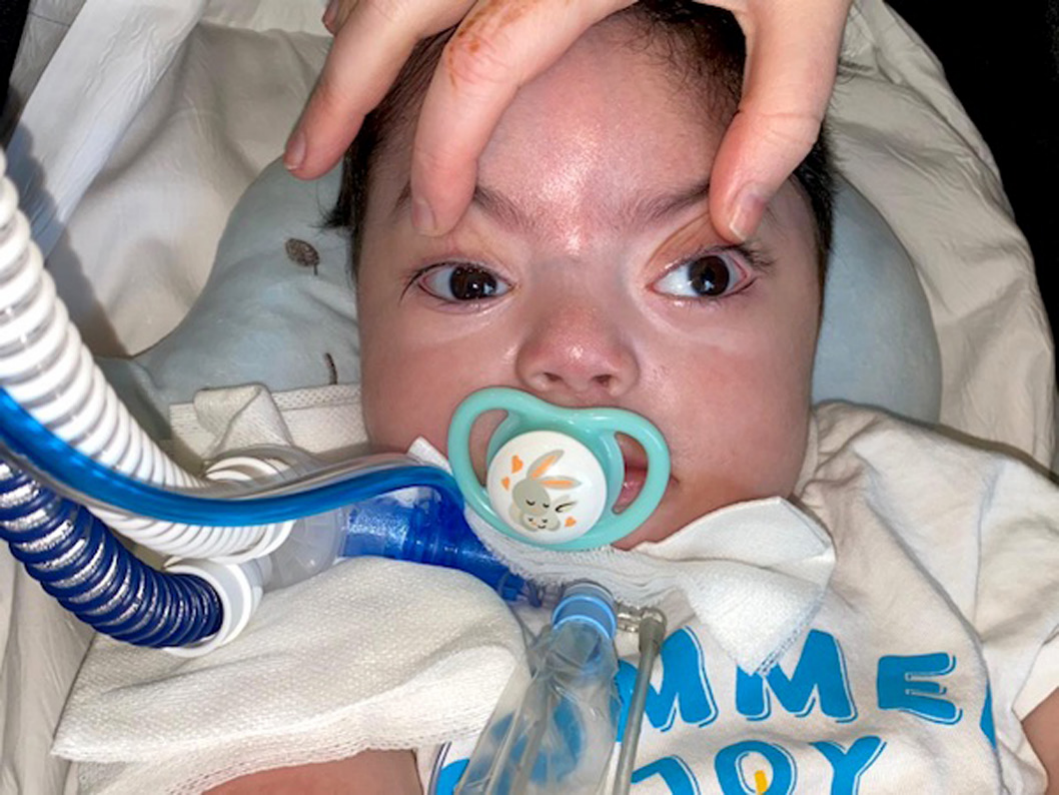
External photograph showing bilateral ptosis from hypotony with exotropia in primary gaze that was transiently larger on attempted saccades to either side.

**FIGURE 4:**
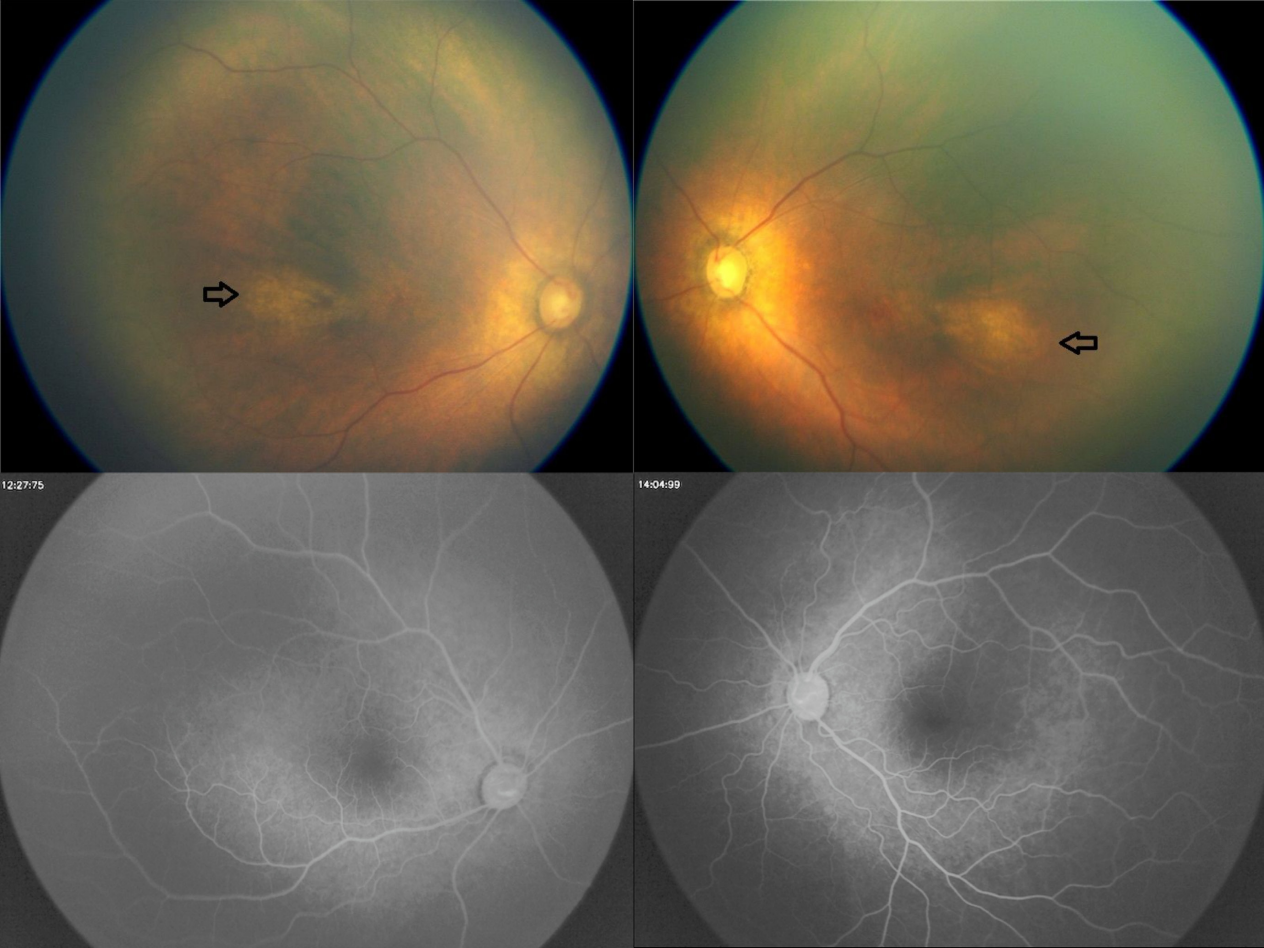
Ocular fundus. Upper panel left—right eye fundus photography showing torpedo‐like maculopathy changes (black arrow) and increased cupping. Upper panel right—left eye fundus photography showing torpedo‐like maculopathy changes (black arrow) with increased cupping. Lower panels—Fundus fluorescein angiogram showing a mild hyperfluorescence without leakage in both eyes.

He had bilateral ptosis with infrequent and incomplete blinking bilaterally in early infancy that may have improved somewhat as he aged. He had exotropia in primary gaze that was transiently larger on attempted saccades to either side in a fashion that might imply a drift of both eyes on adduction (Figure 3). Nevertheless, he was able to fix and follow horizontally to either side, and he had no pathologic spontaneous eye movements. Corneas, conjunctivae, anterior chambers, and pupils appeared normal. Visual acuity could not be quantified given the child's very young age and limited responsiveness. He had torpedo‐like maculopathy changes in both eyes with increased cupping of the optic disks. Fundus fluorescein angiogram showed mild hyperfluorescence without leakage in both eyes (Figure 4).

Whole‐genome sequencing performed after 2 weeks of life revealed homozygous variants c.1379C>T(p.S460L) in the ZNF699 gene on chromosome 19p13.2. This variant is associated with developmental delay and variable developmental abnormalities in multiple organ systems known as DEGCAGS, similar to the presentation of this child (Bertoli‐Avella et al., 2021; Biela et al., 2022). He also had a homozygous 10 kb deletion on chromosome 15 affecting exons 4 to 8 of the TGM7 gene and a homozygous variant c.869C>T(p.A290V) in the ALG12 gene, which has no known clinical sequelae. Neither of these novel abnormalities in TGM7 or ALG12 genes are currently recognized to cause otherwise typical developmental delay, hypotonia, or the other developmental abnormalities reported previously with these genes.

The child subsequently remained clinically stable without improvement in hypotonia or developmental status. From NICU, he was transferred to a long‐term care facility with follow‐up provided by a team consisting of a neurologist, ophthalmologist, geneticist, dietician, rehabilitation physician, and other medical personnel as necessary. Following discussion with the hospital's ethical committee and the institutional review board, this report was exempt from review. The consent for publication has been obtained from parents.

## 3 DISCUSSION

We describe the 15th child to be presumably identified with the DEGCAGS syndrome and the first individual with homozygous missense variants in the ZNF699 gene who had complete clinical examination and detailed retinal imaging (Table 1). While there is insufficient evidence to classify the variant as pathogenic, this novel variant is rare for a recessive disorder (not present in the Franklin ACMG classification in any homozygous state: maximal non‐founder subpopulation frequency: 0.042%). In 2021, Bertoli‐Avella et al. (2021) described 13 patients from 12 consanguineous Middle Eastern families with homozygous loss‐of‐function insertions or deletions in the ZNF699 gene discovered after whole genome/exome sequencing of 33,000 individuals with developmental delay. In 2022, Biela et al. (2022) recognized the syndrome on clinical grounds for the first time in an infant with compound heterozygous mutations in the same gene. Including the patient reported here, our current knowledge of the DEGCAGS syndrome focuses attention on neurodevelopmental delay (present in all 15 patients) together with other serious medical problems implying malfunctioning of other organ systems (pulmonary, GI, immune) and threatening life.

**TABLE 1:** Genetic variants in the ZNF699 gene reported causing DEGCAGS syndrome.

Variant	NCBI	Zygosity	Report
NM_198535.2: c.436‐439del	Human hang ortholog	Homozygous	Bertoli‐Avella et al. (2021)
NM_198535.2: c.1623‐1636delTTAT	Human hang ortholog	Homozygous	Bertoli‐Avella et al. (2021)
NM_198535.2: c.1324dupA	Human hang ortholog	Homozygous	Bertoli‐Avella et al. (2021)
NM_198535.2: c.349dupA	Human hang ortholog	Homozygous	Bertoli‐Avella et al. (2021)
NM_198535.2: c.51_54delCTCA	Human hang ortholog	Homozygous	Bertoli‐Avella et al. (2021)
NM_198535.3: c.535C>T, c.1327C>T	Human hang ortholog	Compound heterozygous	Biela et al. (2022)
NM_198535.3 exon 6 c.1379C>T p.S460L	Human hang ortholog	Homozygous in exon 6	Current article

Abbreviations: DEGCAGS, developmental delay with gastrointestinal, cardiovascular, genitourinary, and skeletal abnormalities; NCB, National Center for Biotechnology Information.

The most consistent hallmark of the DEGCAGS syndrome is severe neurodevelopmental delay, which is shared by all 15 currently recognized patients. Many of the reported DEGCAGS patients had severe hypotonia, globally decreased movement with normal DTRs, and no obvious spasticity. Bertoli‐Avella et al. (2021) reported abnormal brain MRI in two of their 13 patients, one with agenesis of the corpus callosum and another with hypoplastic cerebellar vermis and delayed myelination; four other patients were reported to be microcephalic. The patient reported by Biela et al. (2022) had ventricular enlargement on computed tomography and some hyperintensity of periventricular white matter on ultrasound. Our patient had no gross brain injury or malformation on neuroimaging severe enough to explain his hypotonia and decreased mobility. Thus, DEGCAS is a profound, variable, congenital, non‐progressive syndrome causing significant central nervous system abnormalities and also affecting multiple other organ systems (Table 2). It is associated to date with homozygous or compound heterozygous variants in the ZNF699 gene, a zinc‐finger gene on chromosome 19.2 that encodes a large nuclear protein that may well have a role in nucleic acid binding (Biela et al., 2022). The spectrum of clinical features appears similar in individuals with insertions or deletions of the ZNF699 gene or missense variants.

**TABLE 2:** Congenital clinical problems in DEGCAGS syndrome.

Problem	Bertoli‐Avella et al. (2021)	Biela et al. (2022)	Current report
Neurodevelopmental delay	13/13	1/1	Yes
Intrauterine growth retardation	5/13		Yes
Polyhydramnios	5/13		No
Premature birth	5/13		Minimally
Hypotonia	7/13		Yes
Recurrent infections	5/13		Yes
Anemia	6/13		Yes
Feeding difficulties	4/13		Yes
Gastrointestinal anomalies	7/13	Intestinal atresia	No
Cardiovascular anomalies	2/13	No	PFO
Genitourinary anomalies	3/13		Bilateral cryptorchidism
Skeletal anomalies	Syndactyly in 7/13		Syndactyly, contractures of elbows and knees
Respiratory anomalies		Resuscitation	Resuscitation

Abbreviation: DEGCAGS, developmental delay with gastrointestinal, cardiovascular, genitourinary, and skeletal abnormalities.

It is not currently known whether homozygous ZNF699 mutations might occasionally cause other symptoms in the peripartum period or other clinical presentations at older ages. Some reported patients had intrauterine growth retardation (6/15), polyhydramnios (6/15), premature birth (6/15), and failure to thrive (Table 2). All patients had facial or/and skeletal dysmorphisms, including abnormal facial shape (10/15), microcephaly (6/15), long eyelashes (4/15), abnormal eyebrows (4/15), and an abnormal nose (5/15). Less common facial abnormalities included hypertelorism, low‐set ears, microphthalmia, and ptosis. The child reported here was delivered at 37 weeks gestation because of placental insufficiency and breech presentation, and in the peripartum period, he had low APGAR scores, hypotonia with minimal spontaneous movements, hypoxia, and hypotension. He had facial dysmorphia and skeletal malformations and required resuscitation and emergency management including a tracheostomy, a gastrostomy tube, and antibiotics several times because of presumed sepsis. It is unclear whether hypotonia gets better with age as the oldest documented patient is 16 months of age.

Until this point, little has been known about the visual system in DEGCAGS patients. Some individuals were noted to have hypertelorism, ptosis, and/or proptosis, and Bertoli‐Avella et al. (2021) reported microphthalmia in two patients and an abnormal ERG in one. The child reported here had bilateral partial ptosis and some limitation of adduction OU, possibly implying a synaptic problem. The retinal examination in the DEGCAGS syndrome has not been described previously, but the child reported here had modest optic disk cupping and a torpedo‐like maculopathy bilaterally. Currently, we do not know how frequent visual abnormalities might be in patients with homozygous ZNF699 variants. While the role of homozygous variants in TGM7 ALG12 genes present in this patient seems unclear at this point, we acknowledge that novel TGM7 variant could be a good candidate for this clinical presentation since there is no known disease associated with this gene and no homozygous loss of function in The Genome Aggregation Database (gnomAD).

Zinc‐finger proteins interact with DNA, RNA, and other proteins. They are implicated in regulation of transcription, DNA repair, ubiquitin‐mediated protein degradation, signal transduction, actin targeting, cell migration, and numerous other processes (Cassandri et al., 2017). Mutations in other zinc‐finger proteins have been reported in association with neurologic problems similar to those described here. For example, ZNFs have a regulatory function in synaptic and muscle differentiation (Cassandri et al., 2017). The KDM5 gene contains a C5HC2 zinc finger, and a mutation in this motif is associated with ID and reduces NMJ bouton number, increases bouton size, and alters microtubule dynamics (Belalcazar et al., 2021). Several genes encoding ZnF proteins have been implicated in other human pathologies including nonsyndromic X‐linked mental retardation (Lugtenberg et al., 2006; Moreira et al., 2001; Shoichet et al., 2002), and Dandy–Walker malformation (Grinberg et al., 2002).

## AUTHOR CONTRIBUTIONS

Syed M. Ali, Dua A. AlMasri, and Igor Kozak contributed to the concept. Syed M. Ali and Dua A. AlMasri contributed to the data collection. Igor Kozak, Carlos E. Prada, Doris Lin, and Thomas M. Bosley contributed to the data analysis. Igor Kozak, Carlos E. Prada, Doris Lin, and Thomas M. Bosley contributed to the manuscript writing. Igor Kozak, Carlos E. Prada, Doris Lin, and Thomas M. Bosley contributed to the manuscript review/revision.

## CONFLICT OF INTEREST STATEMENT

None.

## ETHICS STASTEMENT

Following discussion with the hospital Ethical Committee and the Institutional review Board of the Moorfields Eye Hospital and Health Plus, this report was exempt from review. The consent for publication has been obtained from parents. All procedures were performed in accordance with the relevant ethical standards.

## Data Availability

The data that support the findings of this study are available on request from the corresponding author. The data are not publicly available due to privacy or ethical restrictions.
